# Effect of oxygen on the per‐cell extracellular electron transfer rate of *Shewanella oneidensis* MR‐1 explored in bioelectrochemical systems

**DOI:** 10.1002/bit.26046

**Published:** 2016-07-21

**Authors:** Mengqian Lu, Shirley Chan, Sofia Babanova, Orianna Bretschger

**Affiliations:** ^1^ Department of Microbial and Environmental GenomicsJ. Craig Venter Institute4120 Capricorn LaneLa JollaCalifornia 92037; ^2^ Chemical and Biological EngineeringUniversity of New MexicoAlbuquerqueNew Mexico

**Keywords:** *Shewanella oneidensis* MR‐1, per‐cell EET rate, oxygen, flavin

## Abstract

Extracellular electron transfer (EET) is a mechanism that enables microbes to respire solid‐phase electron acceptors. These EET reactions most often occur in the absence of oxygen, since oxygen can act as a competitive electron acceptor for many facultative microbes. However, for *Shewanella oneidensis* MR‐1, oxygen may increase biomass development, which could result in an overall increase in EET activity. Here, we studied the effect of oxygen on *S. oneidensis* MR‐1 EET rates using bioelectrochemical systems (BESs). We utilized optically accessible BESs to monitor real‐time biomass growth, and studied the per‐cell EET rate as a function of oxygen and riboflavin concentrations in BESs of different design and operational conditions. Our results show that oxygen exposure promotes biomass development on the electrode, but significantly impairs per‐cell EET rates even though current production does not always decrease with oxygen exposure. Additionally, our results indicated that oxygen can affect the role of riboflavin in EET. Under anaerobic conditions, both current density and per‐cell EET rate increase with the riboflavin concentration. However, as the dissolved oxygen (DO) value increased to 0.42 mg/L, riboflavin showed very limited enhancement on per‐cell EET rate and current generation. Since it is known that oxygen can promote flavins secretion in *S. oneidensis*, the role of riboflavin may change under anaerobic and aerobic conditions. Biotechnol. Bioeng. 2017;114: 96–105. © 2016 The Authors. *Biotechnology and Bioengineering* Published by Wiley Periodicals, Inc.

## Introduction

Microbial bioelectrochemical systems (BESs) use microbial catalysts at electrode surfaces to convert chemical energy into electric energy (Kelly and He, 2014; Rosenbaum and Franks, 2014). Common BESs include microbial fuel cells (MFCs), microbial electrolysis cells (MECs) and microbial electrosynthesis (MES) etc., (Rosenbaum and Franks, 2014) which have shown potential applications for wastewater treatment, biosensing, and chemical production. The bacteria at BES anodes oxidize organic substances and move the electrons released during the oxidation process through their biological electron transport chain to the surface of electrodes in a process termed extracellular electron transfer (EET). One of the key challenges for BES development as power sources is to maximize the electricity output by maintaining specific environmental parameters, such as pH, dissolved oxygen (DO) concentration, temperature, salinity, organic feed, and electrode material (Clauwaert et al., 2008; Fan et al., 2007; Logan et al., 2007). Oxygen, even at trace amounts, has been considered detrimental to BES performance by competing with the anode for the electrons and thus decreases the overall Coulombic efficiency (Fan et al., 2007; Liu and Logan, 2004; Logan et al., 2006; Min et al., 2005; Quan et al., 2012; Ringeisen et al., 2007; TerAvest et al., 2014). Oxygen can also possibly be toxic to minimally aerotolerant and strictly anaerobic electro‐active bacteria (Freguia et al., 2008; Lin et al., 2004; TerAvest et al., 2014). Therefore, the anodes in BESs are commonly maintained under anaerobic conditions.

*Shewanella*, as a model microbe, uses multiple electron transfer strategies, including direct electron transfer and mediated electron transfer. Direct electron transfer occurs through direct contact between the outer‐membrane and solid electron acceptors like iron and manganese oxides, or through conductive extended nanowires, which have been demonstrated to be extensions of the outer membrane and periplasm (Pirbadian et al., 2014). Mediated electron transfer occurs through electron shuttles, such as self‐secreted flavins. It has been also demonstrated that flavin compounds interact with the outer‐membrane (OM) *c*‐type cytochromes (c‐Cyts) MtrC and OmcA to enhance the EET rate (Okamoto et al., 2014; Xu et al., 2016). *Shewanella spp*. are also well‐known as facultative exoelectrogens that can grow and produce power under both anoxic and oxic conditions (Biffinger et al., 2008, 2009; Quan et al., 2012; Rosenbaum et al., 2010). Utilizing the aerotolerance property of *Shewanella* in BES is an attractive option since it can possibly recover electricity from organic substrate without the need to maintain an extremely anoxic condition (Biffinger et al., 2008). However, studies on the effect of oxygen on current production of *Shewanella* in BESs have shown inconsistent results. Generally, oxygen is considered to be detrimental, while adjusting BES configurations can lessen the effects of oxygen as a competitive electron acceptor. Ringeisen et al., have shown that using miniaturized MFCs to enhance proton diffusion to cathodes can enable electricity generation by *Shewanella* even at saturated DO concentrations (Ringeisen et al., 2007). Other research results showed that oxygen exposure at certain levels can increase the current production in *Shewanella* BESs (Biffinger et al., 2009; Rosenbaum et al., 2010; TerAvest et al., 2014).

Multiple reasons have been proposed to explain the increased current output with oxygen exposure in *Shewanella* pure‐cultured BESs: (i) Oxygen allows more efficient substrate utilizations (Biffinger et al., 2008; Rosenbaum et al., 2010); (ii) Oxygen enhances nicotinamide adenine dinucleotide (NADH) production and thus boosts the charge delivered to the anode (Li et al., 2010); (iii) Oxygen may promote *S. oneidensis* self‐secretion of mediators, such as flavins to aid current production (TerAvest et al., 2014); (iv) Oxygen enhances biomass growth, and therefore increases the number of electrochemically active cells on the electrode (Biffinger et al., 2009; Liu et al., 2015; Rosenbaum et al., 2012, 2010; TerAvest et al., 2014). To clarify how oxygen affects the electricity generation, it is necessary to separate the effect of biomass growth and evaluate the per‐cell performance. However, it is difficult to study the biomass change and associated current production in real‐time within most BES reactors since it is not practical to regularly remove biomass from an operating BES for quantification, and unfeasible to run multiple BESs under exactly identical conditions and terminate them one by one for electrode sampling. Therefore, the effect of oxygen on *Shewanella* electricity generation over time and under different BES configurations is still unclear.

Here, we use optically accessible BES designs to elucidate the effects of oxygen on *S. oneidensis* MR‐1 electricity generation by separating the influence of factors such as biomass growth, per‐cell EET rate change, and BES configuration/operation. The optically accessible BES design allows real‐time noninvasive monitoring of cell populations and thus the calculation of per‐cell EET rate (McLean et al., 2010). Our experiments indicate that oxygen exposure always promotes biomass growth and impedes per‐cell EET rate. The oxygen effect on electricity generation is the result of competition between the increased cell population and decreased per‐cell EET rate. At a certain level of oxygen exposure (for example, 0.2 mg/L DO in our experiment), the increase in biomass overcomes the decrease in per‐cell EET rate, resulting in higher current production than current generated under the anaerobic condition. Our preliminary result showed that higher current density can be achieved by maintaining high oxygen concentration at the starting stage to increase the electrochemically active cells, and removing oxygen later to recover the per‐cell EET rate. Additionally, we found that the effect of riboflavin in enhancing EET rate and current density weakens with oxygen exposure, indicating that the role of riboflavin as electron shuttles can be affected by oxygen. These results were further validated in different BES reactor configurations under batch and flow conditions.

## Materials and Methods

### Growth and Cultivation of Microorganisms

For each experiment, 0.5 mL of *S. oneidensis* MR‐1 p519nGFP (constitutively expressed Green Fluorescent Protein) (MR‐1) frozen stock was added to 100 mL Luria–Bertani broth with 85 μM Kanamycin sulfate, and grown at 30°C with 150 RPM agitation until the optical density (OD_600_) of the culture was 1.0; equivalent to a cell density of approximately 4.0 × 10^9^ cells/mL at 12 h. A minimal medium described in McLean et al. (2010) with 20 mM lactate, 85 μM Kanamycin sulfate was used for subsequent experiments. Riboflavin at different concertation was added to the minimal medium as noted in the study of riboflavin effect on current production.

### Optically Accessible Microbial Fuel Cell (opti‐MFC)

**Figure 1:**
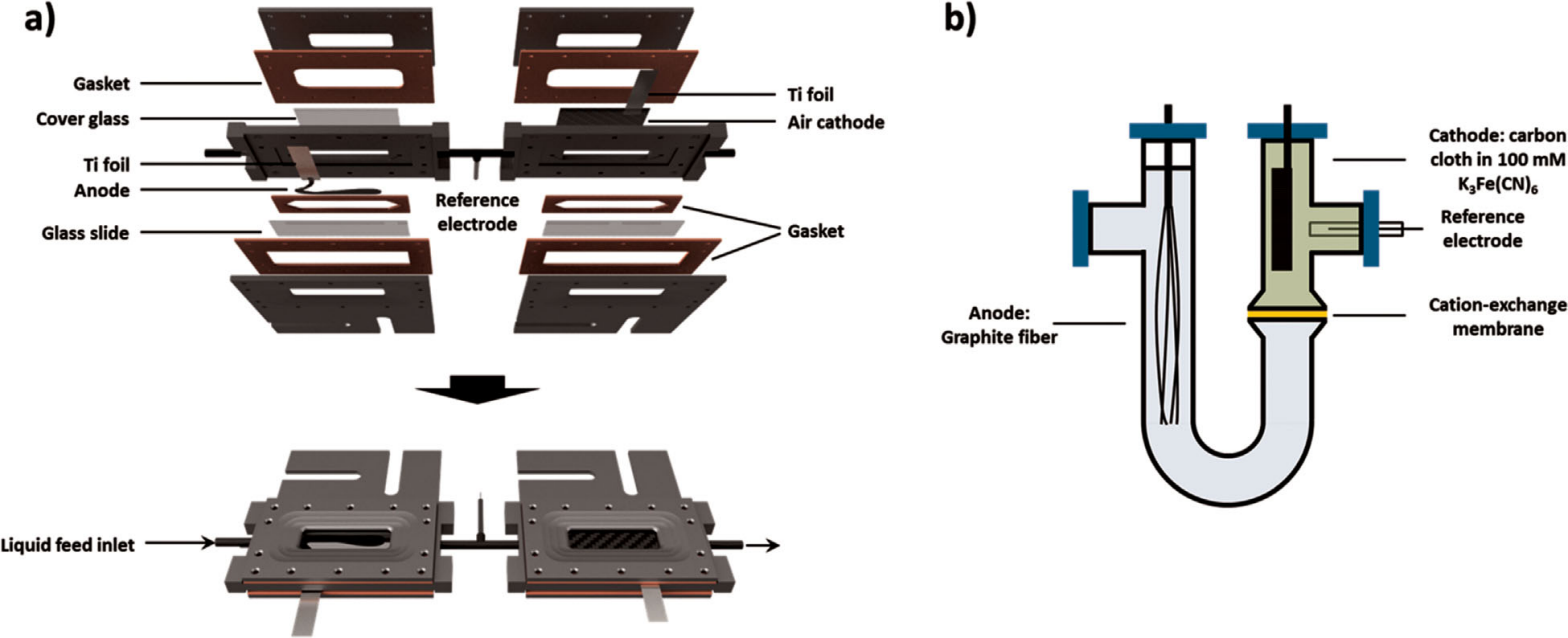
The schematic diagram of (**a**) the optically accessible microbial fuel cell (opti‐MFC) and (**b**) the U‐tube microbial fuel cell (U‐tube).

Optically accessible microbial fuel cells (opti‐MFCs) were constructed similar to previous reports (McLean et al., 2010), with some system modifications (Fig. 1a). The anode and cathode chambers were connected in series such that the medium first entered the anode chamber and then the cathode chamber. The volume of each chamber is 2.8 mL. Ag/AgCl reference electrode (Re‐5B, BASi Corp., West Lafayette, IN) was placed between the anode and cathode chambers. The MFC was operated with a 220 Ω external resistor and the voltage drop across the resistor was recorded every 10 min (Keithley 270, Keithley Instruments, Inc., Beaverton, OR). Current (*I*) was calculated from the measured cell voltage (*V*) and applied resistance (*R*), according to Ohms Law (*V = IR*).

The anode consisted of a single, 6 cm tow of graphite fibers (FG‐CF 19750, US Composites, Inc., West Palm Beach, FL) containing 12,000 individual graphite fibers of 7 μm diameter. The anode was exposed to 100% acetone for overnight, then rinsed and stored in DI water until use. The air cathodes were constructed in the manner described by Liu et al. (HaoYu et al., 2007; Liu and Logan, 2004), which consisted of Pt (5 g/m^2^) coated graphite felt (GDE LT 120E‐W, Fuel Cell Store, College Station, TX), painted with Nafion (Sigma–Aldrich Corp., St Louis, MO) on the surface and allowed to air‐dry. Titanium foils (35881, Alfa Aesar, Ward Hill, MA) were used as leads to make electrical connections in both of the anode and cathode chambers.

Prior to inoculation, the anode chambers of the opti‐MFCs were chemically sterilized with a 10% peroxyacetic acid solution and flushed with minimal medium until a stable baseline voltage was recorded (10 min). Then 10 mL of the pre‐grown MR‐1 culture (OD_600_ = 1.0) was injected and allowed to sit in batch for 10 min, and then was flushed at 4.7 mL/min (hydraulic retention time = 0.6 min) for 10 min. The flow rate was then decreased to 0.3 mL/min (hydraulic retention time = 9.3 min) during operation. The medium reservoir was continuously purged with ultra‐high purity nitrogen unless oxygen concentrations were being evaluated. All tubing was made of Viton to prevent oxygen intrusion through medium delivery. The temperature of each experiment was maintained at 25°C.

### Optically Accessible Poised‐Potential Bioelectrochemical System (opti‐BES)

The optically accessible poised‐potential bioelectrochemical system (opti‐BES) was similar to the opti‐MFC, but without the cathode chamber. The working electrode was the anode in the opti‐MFC; however, the fiber length was reduced from 6 to 4 cm to avoid contact with the counter electrode that was embedded in the same chamber. The counter electrode was a titanium foil with thickness of 0.03 mm and size of 2.5 × 1.0 cm^2^ (7440‐32‐6, Alfa Aesar). A reference electrode was placed just before the liquid feed inlet. The sterilization, inoculation, and operation of the opti‐BES is the same as that mentioned in opti‐MFC section, except the working electrode was poised at +0.2 V versus Ag/AgCl using a potentiostat (Gamry Reference 600, Gamry Instruments, Inc., Warminster, PA), and the current production was monitored through the potentiostat without using external resistor.

### U‐Tube Microbial Fuel Cell (U‐Tube)

The U‐tube microbial fuel cell (U‐tube) was constructed from a U‐shaped tube for the anode chamber (30 mL) and a straight tube that formed the cathode chamber (10 mL) (942993, Adams & Chittenden Scientific Glass, Berkeley, CA), similar to previous reports (Xing et al., 2010). The two chambers were separated by a cation‐exchange membrane (CEM) (1.8 cm^2^; CMI‐7000, Membranes International Inc., Ringwood, NJ) and were joined together by a C‐type clamp (Fig. 1b), sealed with silicone gasket (DSP4038GP‐032, Diversified Silicone Products, Inc., Santa Fe Springs, CA) and silicone sealant. The anode was the same 6 cm fiber electrode as in the opti‐MFC. The cathode was made of 5.0 × 3.5 cm^2^ plain weave carbon cloth (PW06, ZOLTEK Corp., Bridgeton, MO). Titanium wires were used as leads to make electrical connections. The U‐tube was operated with a 220 Ω external resistor and the voltage drop over the resistor was monitored every 10 min using a Gamry multichannel potentiostat.

The U‐tubes were autoclaved and placed in an anaerobic chamber before inoculation. A 100 mM of K_3_Fe(CN)_6 ­_was filtered (0.2 μm) and degassed (20% CO_2_ and 80% N_2_), and then used as catholyte solution. Thirty mililitre of the pre‐grown MR‐1 culture (OD_600_ = 1.0) was injected into the anode compartment and allowed to sit in batch for 10 min. Then the medium and inoculum were replaced with 10 mL of degassed minimal medium with gentle shaking. Then the wash medium was removed and 30 mL of degassed minimal medium was injected as the anolyte solution. The temperature of each experiment was maintained at 30°C and all medium replacements occurred under anoxic conditions.

### Control of Dissolved Oxygen (DO) Concentration

To maintain anaerobic conditions in the opti‐MFC and opti‐BES, the minimal medium was purged with 100% nitrogen for 10 h prior to use and the purging continued during the experiment. This conventionally prepared anaerobic minimal medium still contained trace amount of DO (∼0.08 mg/L), which is sufficient for GFP maturation (Fig. S3) (Hansen et al., 2001). For experiments requiring controlled DO concentrations, highly purified air and nitrogen were both purged into the medium reservoir, while their respective pressures were adjusted separately until the target DO value was reached and stable for 1 h. The DO value of the minimal medium before entering the opti‐MFC or opti‐BES was continuously monitored using a DO probe (LDO10101, Hach Company, Loveland, CO). The DO probe was sterilized with 70% aqueous ethanol solution and rinsed with sterilized deionized water before use.

### Laser Scanning Confocal Microscope and Average Per‐Cell EET Rate

A laser scanning confocal (LSC) microscope (Leica TCS SP5, Leica Microsystems, Buffalo Grove, IL) was used to image cell attachment and biomass growth. A 488 nm laser was used to excite the constitutively expressed green fluorescent protein of the *S. oneidensis* MR‐1 p519nGFP. Every 2 h, LSC microscope images were taken within a given field of view with a *z* depth of about 150 μm. Within the *z* depth, over 10 graphite fibers were captured. The field of view was changed 2–3 times throughout each experiment. The average cell density (*ρ*) was calculated by using the number of the cells counted (*N_partial_*) and geometric surface area (*SA_partial_*) of the imaged fibers:

(1) $$\rho =N_{partial}/{SA}_{partial}$$

The total cell number (*N_total_*) was then calculated by Eq. (2):

(2) $$N_{total}={SA}_{total}\times \rho ={SA}_{total}\times N_{partial}/{SA}_{partial}$$

Here, *SA_total_* is the total surface area of the fibers estimated geometrically. An anode consisting of a 6 cm tow of 12,000 graphite fibers with 7 μm diameter has a total surface area of 158 cm^2^, and a 4 cm anode has a total surface area of 105 cm^2^.

The current (*I*) at a given time point was used to calculate the average per‐cell EET rate of *S. oneidensis* MR‐1 (*I_MR1_*) using Eq. (3):

(3) $$I_{MR1}=I/N_{total}$$

### Flavin Concentration Measurement in U‐Tubes

Direct measurement of flavin concentration in the anolyte in U‐tubes might cause errors, since the anolyte is a complex matrix containing intermediate and final products of the substrate oxidation, which may affect the measurement. Therefore, the standard addition method was used (Roy et al., 2014). The anolyte from U‐tubes was filtered (0.2 μm) to remove microbes, and then spiked with riboflavin solution with known concentration (20, 40, 80, 160, and 300 nM). Then the emission at 530 nm of the samples with excitation at 450 nm was measured using microplate reader (FlexStation 3, Molecular Devices, LLC., Sunnyvale, CA). The emission intensities were plotted versus the spiked riboflavin solution concentration, and fitted with a linear function. The absolute value of the *x*‐intercept was taken as the flavin concentration of the tested samples.

### Internal Resistance and Open Circuit Potential Measurement

The internal resistances of the abiotic BESs were measured using electrochemical impedance spectroscopy (EIS). The impedance spectra were acquired with a Gamry potentiostat (Gamry Reference 600) using the frequency range between 1 MHz and 0.01 Hz and a sinusoidal perturbation with 10 mV amplitude. From the impedance data, Nyquist plots were drawn. The distance from the zero and the second intersection gives the internal resistance of the system. (Dewan et al., 2008).

The open circuit potentials (OCPs) were measured 24 h after inoculation and operation. All measurements were done under the anaerobic conditions.

## Results and Discussion

### Effect of Oxygen on Current Production

**Figure 2:**
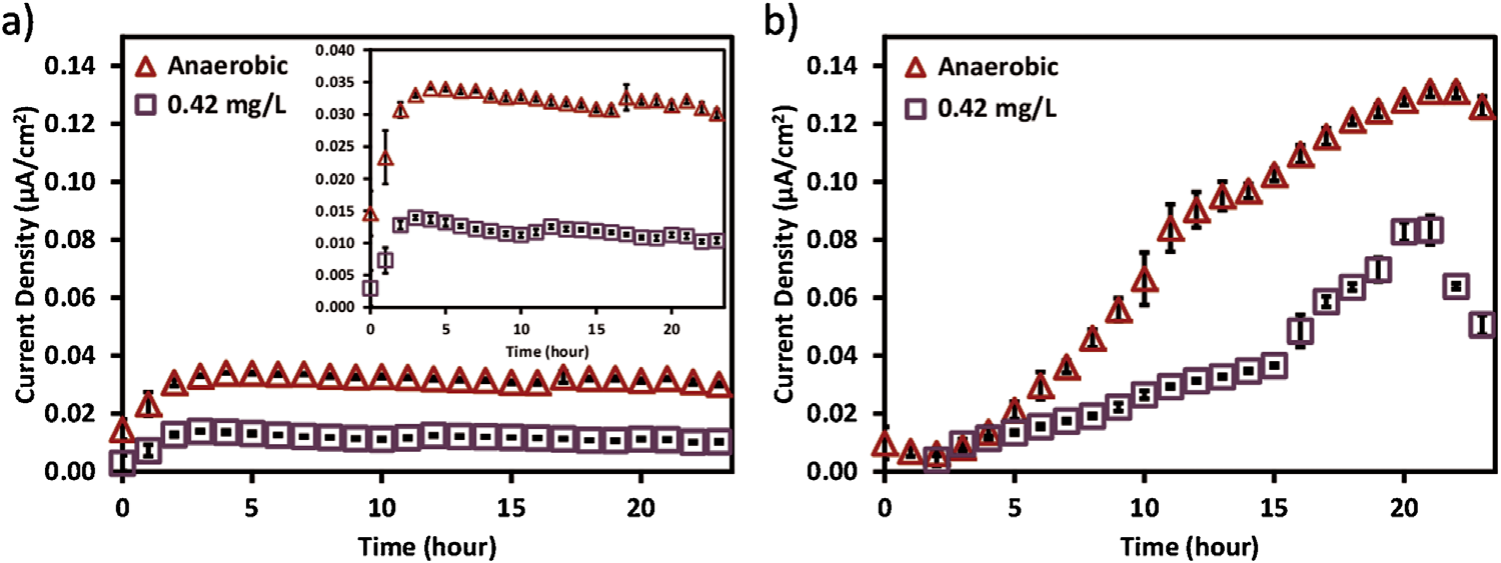
The mean operational current density of (**a**) the opti‐MFC and (**b**) the opti‐BES over 24 h under the anaerobic (triangle) and 0.42 mg/L DO (square) conditions. The inset in (**a**) shows the operational current density in a smaller scale for more details.

We used both opti‐MFC and opti‐BES to investigate the effect of oxygen on the current production from *S. oneidensis* MR‐1. In both opti‐MFC and opti‐BES, oxygen obviously impaired the current production even at DO values as low as 0.42 mg/L (Fig. 2). In opti‐MFC, the maximum current density with 0.42 mg/L DO was 0.014 μA/cm^2^, which was less than half of that under anaerobic conditions (0.034 μA/cm^2^). In opti‐BES, the maximum current density was 0.083 μA/cm^2^ with 0.42 mg/L DO, and 0.13 μA/cm^2^ under anaerobic conditions.

It is noticeable that the maximum current densities in the opti‐BESs were much higher than those in opti‐MFCs. The different performance between the two setups may be due to the differences in internal resistance and the anodic potential (Table I). Because the opti‐BES was constructed in a single chamber, the proton diffusion distance was shorter and the internal resistance was much lower, making electron transfer easier. The working electrode in opti‐BES was poised at +0.2 V (vs. Ag/AgCl) and the measured potential for the counter electrode was 1.00 V, resulting in 0.8 V potential differences. The higher potential differences in opti‐BES also enhanced the electron transfer. Additionally, a more positive applied potential to the working electrode provides higher driving force for substrate oxidation (Wang et al., 2009), and increases theoretical energy yield per equivalent substrate oxidation to promote higher electrochemical activities (Finkelstein et al., 2006). With a more positive potential, the electrode surface shows positive charge, which allows the negatively charged bacteria to adhere easily (Barisci et al., 2003; Rabaey et al., 2007), resulting in higher current output (Carmona‐Martínez et al., 2013; Finkelstein et al., 2006).

**Table I:** The OCPs (vs. Ag/AgCl) and internal resistance (R_int_) of different systems

	OCP_anode_	OCP_cathode_	*R*_int_
opti‐MFC	−0.29 V	0.25 V	41,110 Ω
U‐tube	−0.15 V	0.31 V	441 Ω
	Working electrode potential	Counter electrode potential	R_int_
opti‐BES	0.20 V	1.00 V	70 Ω

The internal resistances were of the abiotic BESs.

The OCPs were measured 24 h after operation.

### Oxygen Promotes Biomass Development and Impairs Per‐Cell EET Rates

**Figure 3:**
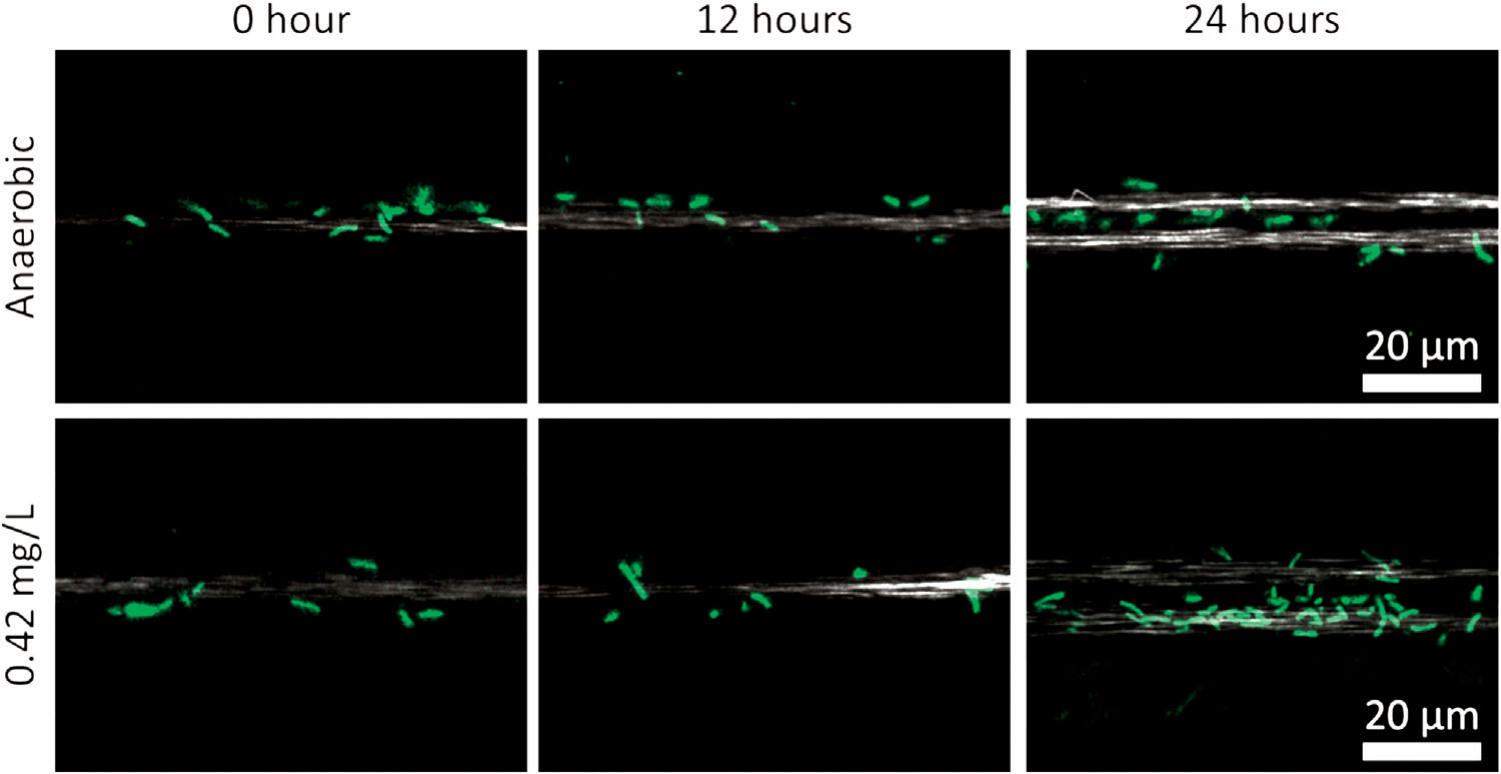
Laser scanning confocal (LSC) microscopy images of GFP‐expressing MR‐1 on anodes in opti‐MFCs at 0, 12, and 24 h, while the MFCs were operated under anaerobic and 0.42 mg/L DO conditions. Under the anaerobic condition, no significant change in cell density after 12 h, while cell growth continued with 0.42 mg/L DO.

The use of opti‐MFC and opti‐BES, allowed real time monitoring of biomass development on electrodes. As shown in Figure 3, in the first 12 h, there was no obvious difference of the cell densities with and without oxygen exposure. Under anaerobic conditions, the cell density was maintained after 12 h with very little biomass apparent at the 24 h time point. However, with 0.42 mg/L DO, the cell density increased after 12 h and showed significantly higher biomass at the 24 h time point. The calculated total cell number confirms this trend (Fig. S1). The cell density difference as a function of DO became more obvious with time. After 120 h, no biofilm had formed under the anaerobic condition, but biofilm development started to show with oxygen exposure (see Fig. S2). It is known that *S. oneidensis* grows slowly under completely anaerobic conditions and that oxygen respiration enables fastest growth (Rosenbaum et al., 2012; TerAvest et al., 2014). Oxygen can also trigger the autoaggregation in *S. oneidensis* by upregulating various attachment and adhesion factors (McLean et al., 2008). Our results confirm that oxygen leads to higher number of *S. oneidensis* MR‐1 on electrodes, which increases the population of electrochemically active cells for current production. However, the higher biomass may not necessarily lead to improved per‐cell EET rates.

**Figure 4:**
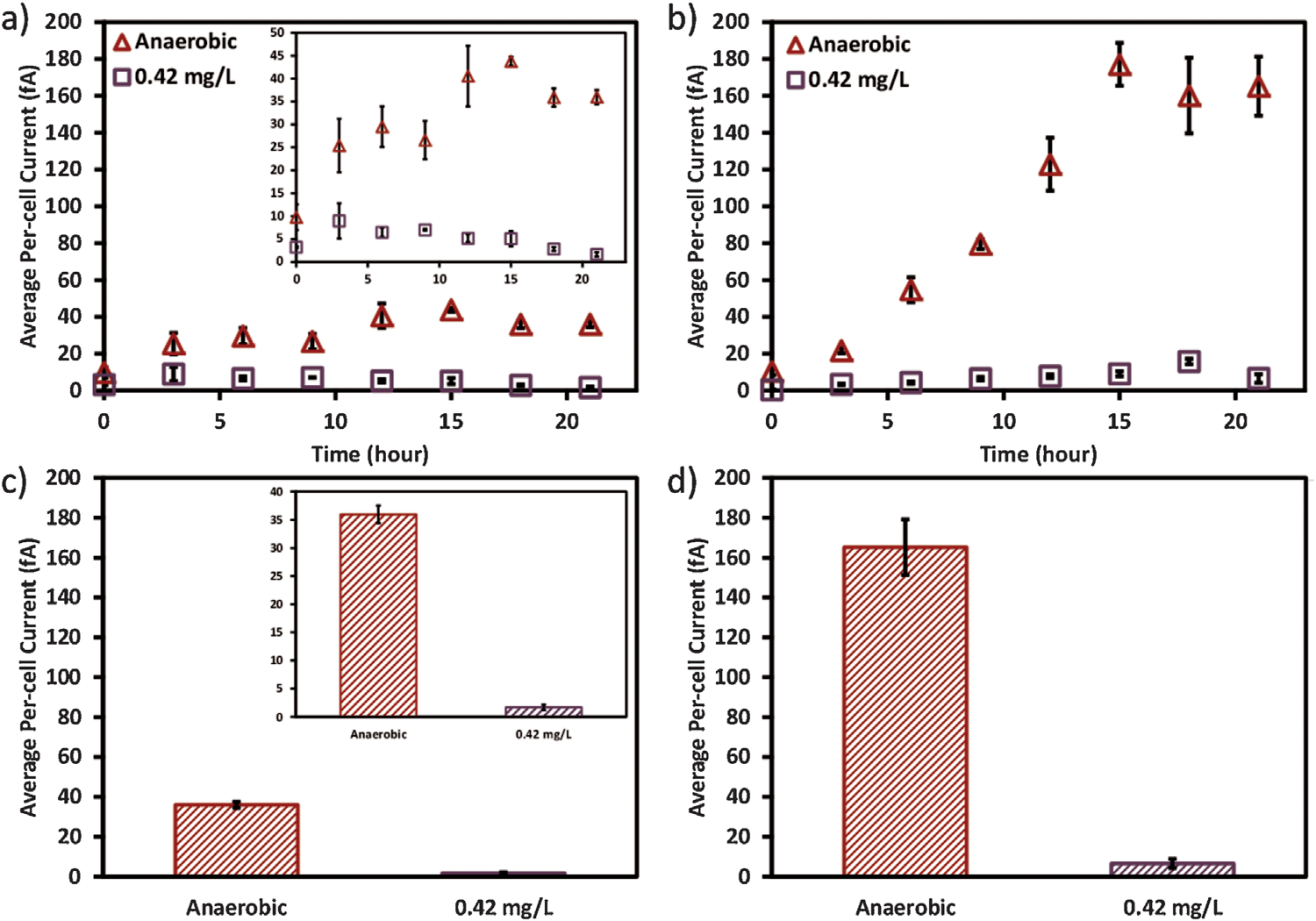
The average per‐cell current of (**a**) the opti‐MFCs and (**b**) the opti‐BES operated under anaerobic (triangle) and 0.42 mg/L DO (square) condition. (**c**) The average per‐cell current at 21 h, when the current densities reached stable values in the opti‐MFC. (**d**) The average per‐cell current at 21 h, when the current densities reached the maximum values in the opti‐BES. The insets in (**a**) and (**c**) show the average per‐cell current in a smaller scale for more details.

The average per‐cell EET rate was calculated using current densities and the total cell number (Fig. 4). In the opti‐MFCs the average per‐cell EET rate increased during the first 3 h under both anaerobic and 0.42 mg/L DO conditions. The average per‐cell EET rate kept increasing until 15 h for the anaerobic condition; however, that the average per‐cell EET rate for the 0.42 mg/L DO condition kept decreasing. The highest average per‐cell EET rate under the anaerobic condition (43.8 fA) was five‐fold higher than that with DO of 0.42 mg/L (8.98 fA). A comparison of the per‐cell current at 21 h when the current densities stabilized showed an even higher ratio between anaerobic and 0.42 mg/L DO conditions (Fig. 4c). Similar trend was also observed in opti‐BES (Fig. 4b and d). However, a higher EET rate was seen in opti‐BESs than that in opti‐MFCs regardless of DO conditions, likely due to the improved capacity of the anode electrode to accept electrons or the decreased internal resistance of the reactor. Overall, in both opti‐MFC and opti‐BES, oxygen exposure impaired the EET rate at per‐cell level.

The per‐cell current of opti‐BES showed a maximum value at around 15 h, and then reached a steady state. This could correspond to the early log phase of *Shewanella* growth, which was also observed in previous studies (Marsili et al., 2010; McLean et al., 2010). Previous results suggest that *Shewanella* slows its EET during log phase growth, possibly because electrons are being diverted to other reservoirs such as cell division and/or reduced organic compound synthesis. Under anaerobic condition, the per‐cell current of opti‐MFC also showed a similar peak. However, in opti‐MFC with 0.42 mg/L DO, no obvious peaks were observed around 15 h. These results might indicate that operational conditions (device configuration and DO value) can affect the growth phase as well as the EET rate.

### Current Production and Average Per‐Cell EET Rate Recovery From High DO

**Figure 5:**
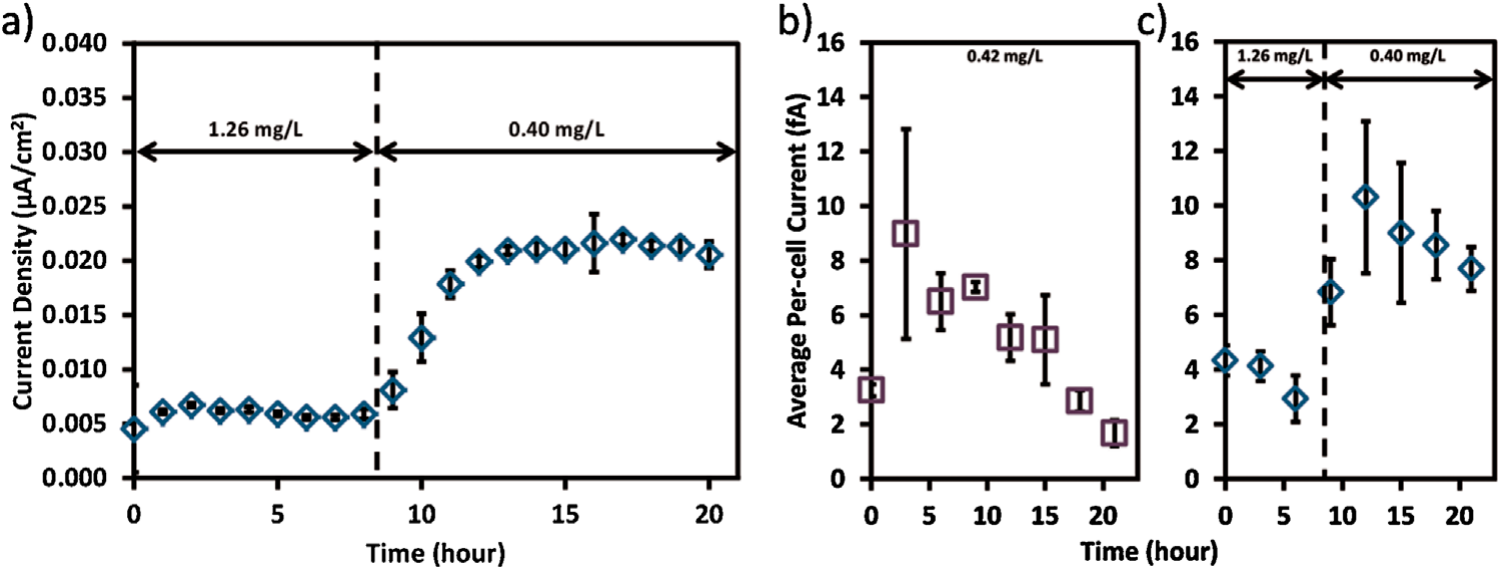
(**a**)The mean operational current density of the opti‐MFC with 1.26 mg/L DO for the first 8.5 h and then 0.40 mg/L DO for the rest of the experiment. The average per‐cell current of the opti‐MFCs with (**b**) 0.42 mg/L DO for 24 h and (**c**) 1.26 mg/L DO for the first 8.5 h and then 0.40 mg/L DO for the rest of the experiment.

As discussed above, oxygen promotes biomass development on the electrode, but impairs the per‐cell EET rate. A simple strategy can be proposed to achieve higher current production, by aerating at the early stages of biofilm formation to enhance biomass development, and decrease oxygen concentration later for higher per‐cell EET rate. Whether *S. oneidensis* MR‐1 can recover the per‐cell EET rate from oxygen exposure determines if this strategy is valid or not. We used opti‐MFC for a preliminary test. The opti‐MFC was aerated with 1.26 mg/L DO for the first 8.5 h and then the DO level was reduced to 0.40 mg/L. When aerated with 1.26 mg/L DO, the maximum current density was only 0.007 μA/cm^2^. Later this low current density gradually increased to 0.022 μA/cm^2^ after the DO decreased to 0.40 mg/L (Fig. 5a). Compared with the opti‐MFC operated at 0.42 mg/L from the beginning (Fig. 2a), the opti‐MFC recovered from high DO value showed 57% higher current production. Also, after the DO level dropped from 1.26 to 0.40 mg/L, the per‐cell current production recovered to a level similar to the opti‐MFCs operated at 0.42 mg/L from the start of the experiment (Fig. 5b), indicating that the per‐cell EET rate can be recovered after DO is decreased. However, the current per cell consistently decreased over time when the DO was maintained around 0.4 mg/L either from the beginning of the experiment, or transitioned from a higher DO. Future work will evaluate why there is a drop in current per cell, as this pattern could indicate a physiological transition taking place within the biofilm (Marsili et al., 2010; McLean et al., 2010). Understanding this transition phase may ultimately lead to greater control over biomass development and improved EET rates.

### Flavin Effect on Current Production

**Figure 6:**
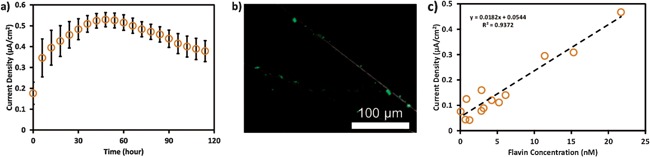
(**a**) The mean operational current density of the U‐tubes. (**b**) LSC microscopy images of the anode taken from U‐tube at 120 h and stained with Sybr green. (**c**) The variation of current density of U‐tubes with the change of flavin concentration.

During the continuous‐fed experiments, even though the medium was purged with nitrogen continuously, still trace amount of oxygen may exist. Batch‐fed experiments without flowing medium may be controlled to be even more strictly anoxic. Therefore, batch‐fed U‐tube microbial fuel cells (U‐tubes) were used to verify the current production of *S. oneidensis* MR‐1 under more strictly anaerobic conditions. The current density of the U‐tubes is shown in Figure 6a, which was an order of magnitude higher than that of opti‐MFCs. The highest current density of the U‐tubes (0.529 μA/cm^2^) was 15‐fold higher than the maximum current density observed for the opti‐MFCs (0.034 μA/cm^2^). However, maximum current densities were observed in the U‐tubes after 48 h, and the opti‐MFCs consistently reached a maximum current density after 4 h of operation.

At the end of the U‐tube experiment (120 h), the anode electrode was removed, stained with Sybr green, and imaged using LSC microscopy. As shown in Figure 6b, the cell density on the U‐tube anode after 120 h was lower than that in the anaerobic opti‐MFCs at 24 h (Fig. 3). The total number of cells attached to the electrode at the end of the U‐tube experiment was estimated to be 2.34 × 10^7^, which was an order of magnitude lower than the total cell number in the anaerobic opti‐MFCs after 24 h. Since, planktonic cells cannot be flushed away in U‐tubes and may contribute to EET through mediated electron transfer (Biffinger et al., 2009; Mao and Verwoerd, 2014), the total electrochemically active cell number cannot be accurately calculated by solely counting the anode‐attached bacteria. Therefore, the average per‐cell EET rate cannot be calculated in this case.

The high current production in U‐tubes may be due to the operational conditions and reactor configurations. First, the U‐tubes were operated at 30°C, and the opti‐MFCs and opti‐BESs were operated at 25°C. However, 5°C difference in operational temperature cannot explain the huge difference in current density, as temperature has limited effect on MFC performance (Liu et al., 2005). Second, the anodic OCP in U‐tubes was −0.15 V and the cathodic OCP was 0.31 V, resulting in 0.46 V in potential difference (Table I), which was even lower than that of the opti‐MFCs (0.54 V). The internal resistance of U‐tubes was 441 Ω, which was also higher than that in opti‐BES (70 Ω). The use of potassium ferricyanide as final electron acceptor may contribute to the high current production in U‐tubes. Additionally, the U‐tubes were operated under batch conditions, so the electrolyte could maintain planktonic cells and secretions, while in the opti‐MFCs and opti‐BESs planktonic cells and mediators were consistently removed by medium replacement.

*S. oneidensis* MR‐1 has been known to have multiple mechanisms for EET through both direct electron transfer (DET) and mediated electron transfer (MET). Riboflavin (RF) and flavin mononucleotide (FMN) are the main electron shuttles that have been reported to transfer electrons between *S. oneidensis* and external electron acceptors (Carmona‐Martínez et al., 2011; Coursolle et al., 2010; Li et al., 2010; Liu et al., 2015; Marsili et al., 2008; Rosenbaum et al., 2010; TerAvest et al., 2014). Previous experiments have shown that flavin‐based MET could contribute up to 80% of the EET from *S. oneidensis* (Marsili et al., 2008). Therefore, we postulate that flavin excretion was a major contributor to the observed high current production in the U‐tubes.

Control experiments were carried out to study the effect of flavin in the U‐tubes. After inoculation and 24 h of operation, the anolyte in the U‐tubes was replaced with fresh minimal medium to remove planktonic cells and flavins. Then the current production was continuously monitored, while the anolyte was sampled every 2 h to quantify flavin concentrations (Fig. 6c). We found that 25 nM flavin (monitored in the bulk solution) can cause a five‐fold increase in current density and the current density measured at this concentration was similar to the maximum current density observed after 48 h of operation in the U‐tube experiments (Fig. 6a).

**Figure 7:**
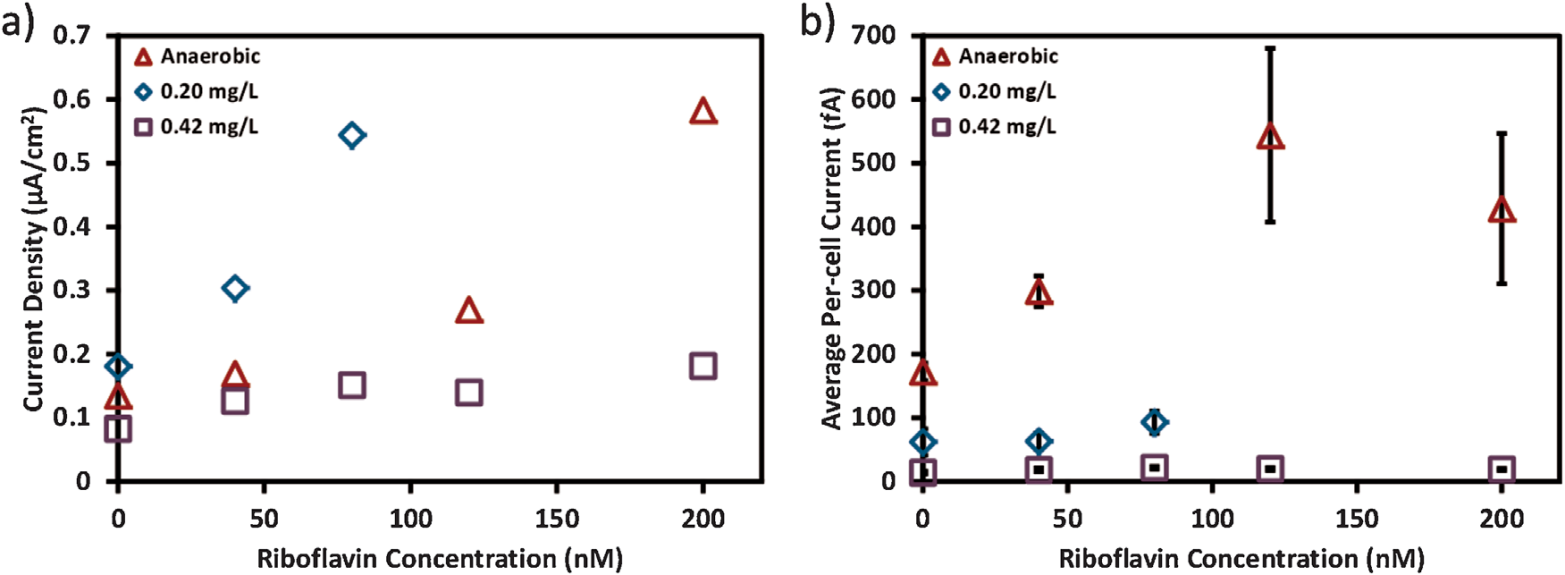
(**a**) The current density and (**b**) the average per‐cell current of the opti‐BES as the riboflavinconcentration changed under anaerobic, 0.2 and 0.42 mg/L DO conditions.

The effect of flavin was again tested in the opti‐BESs under anaerobic, 0.2 and 0.42 mg/L DO conditions. After 24 h of operation, minimal medium with different concentrations of riboflavin was injected into the opti‐BESs until current production reached steady state. The results showed that riboflavin can boost the current production greatly (Fig. 7a). However, the opti‐BESs required much higher concentrations of flavin than in U‐tubes to reach five‐fold improvements in current density, which could be due to several reasons. First, in U‐tubes, the flavin was secreted by the *S. oneidensis* MR‐1, so the flavin concentration within or around the cells could be higher than that in the bulk medium. Thus, the flavin concentration measured in the bulk medium in U‐tubes maybe lower than the actual flavin concentration needed in opti‐BES to reach the same level of increase in current density. Second, riboflavin has been shown to adhere to surfaces and bind to *S. oneidensis* MR‐1 *c*‐type cytochromes (Okamoto et al., 2013, 2014). Therefore, higher concentrations of riboflavin may have been needed under flow conditions to adhere to the electrode surfaces and outer‐membrane proteins under flow conditions.

Interestingly, without riboflavin addition, the 0.2 mg/L DO condition showed a higher current density compared to that under anaerobic condition. Additionally, under 0.2 mg/L DO condition, only 80 nM of riboflavin was needed to increase the current density by five‐fold, while under anaerobic condition, 200 nM of riboflavin was needed to increase the current density by five‐fold. However, the per‐cell current under the 0.2 mg/L DO condition was still much lower than that under anaerobic conditions, indicating that the increase in biomass (Fig. S1c) instead of EET rate contributed to the higher current density at 0.2 mg/L. As oxygen promoted biomass development, a portion of cells may be free floating in medium before reaching and attaching to graphite fibers, making the total cell number possibly higher than the calculated value. The overall effect of oxygen on the current density is the result of the competition between the increased cell population and decreased per‐cell EET rate. Under certain DO level, such as 0.2 mg/L in this case, the increase in biomass can overcome the decrease in per‐cell EET rate, resulting in higher current than the current generated under anaerobic condition. The DO level, which can increase the overall current density, is dependent on the reactor design, reactor volume, carbon source, BES operation, flow rate, flavin concentration, duration of experiments etc. This may elucidate the inconsistency with previous studies regarding the effect of oxygen exposure on current production. As the DO value increased to 0.42 mg/L in the opti‐BES, the decrease in EET rate overcame the increase in biomass, resulting in lower current density compared to the anaerobic condition.

It is also noticeable that the effect of riboflavin on current density varied with oxygen concentration. Under anaerobic and 0.2 mg/L DO conditions, the current density and per‐cell current increased as the riboflavin concentration increased. However, as the DO value reached 0.42 mg/L, riboflavin showed limited effect on either the current density or the per‐cell current. This is possibly due to the effect of oxygen on *c*‐type cytochromes expression and the interactions between flavins and *c*‐type cytochromes. Flavins have been shown to interact with OmcA and MtrC during EET processes (Okamoto et al., 2013, 2014). Recent results from Xu et al. (2016) have also suggested that secreted free‐flavins do not contribute to EET from MR‐1 to electrodes and that only cytochrome‐bound flavins function as redox cofactors. However, oxygen exposure can lower the expression of genes encoding MtrABC and OmcA (Beliaev et al., 2002; Rosenbaum et al., 2012) and thereby limit the number of *c*‐type cytochromes available for riboflavin to interact with. Additionally, oxygen exposure can reverse the binding between MtrC and flavins, and release any bound flavin to the medium (Edwards et al., 2015). Therefore, a DO value high enough to cause changes in *c*‐type cytochromes expression and their interaction with flavins can limit the effect of riboflavin on EET rate and current production. However, even though the role of flavins as electron shuttles can be weakened by oxygen, oxygen exposure can enhance flavin secretion in *S. oneidensis* (TerAvest et al., 2014), indicating that flavins might have different functions under aerobic and anaerobic conditions. Future studies are needed to understand the role of flavins under different DO conditions.

Three‐factor ANOVA tests were performed to verify the effect of experimental factors on current density, total cell number and per‐cell EET rate (Table II), using data at 21 h of operation. The result indicated that oxygen does not show significant effect on current density, but can strongly affect the total cell number and the per‐cell current. Riboflavin does not show significant effect on total cell number, and its effect on per‐cell current and current density is also not significant, probably due to the limited enhancement observed under 0.42 mg/L DO condition. These results again agree with the earlier conclusion that oxygen consistently decreases the per‐cell EET rate, but does not always decrease the overall current density. Riboflavin can enhance the per‐cell current and current density, but the enhancement will be affected by oxygen.

**Table II:** Summary of *P* values from three‐factor ANOVA tests using current density, total cell number or average per‐cell current as the response variables

	Current density	Total cell number	Average per‐cell current
Factors			
A: Oxygen concentration (0, 0.20, 0.42 mg/L)	1.42e‐01	5.48e‐09^***^	7.76e‐06^***^
B: opti‐MFC versus opti‐BES	1.42e‐03^**^	1.26e‐01	9.30e‐03^**^
C: Riboflavin concentration (0, 40, 80, 120, 200 nM)	1.33e‐02^*^	8.56e‐02	1.59e‐03^**^
Interactions			
A:B	3.31e‐01	4.55e‐01	2.34e‐02^*^
A:C	4.07e‐02^*^	1.76e‐01	1.26e‐02^*^
B:C	1.16e‐03^**^	4.51e‐01	2.21e‐05^***^

The same factors (as listed) were used in each test. Significance codes for *P* values:

^*^ ≤5.00e‐02.

^**^ ≤1.00e‐02.

^***^ ≤1.00e‐03.

## Conclusions

We independently studied the effect of oxygen on biomass development and per‐cell EET rates for the facultative exoeletrogen *S. oneidensis* MR‐1 by using opti‐MFC, opti‐BES, and U‐tubes. Our results showed that oxygen promotes biomass development on electrodes and thus increases the number of electrochemically active cells. Meanwhile, oxygen scavenging by *S. oneidensis* MR‐1 reduces the per‐cell EET rate under all conditions tested. The overall effect of oxygen on current production was a competition between increased biomass and decreased per‐cell EET rate.

Our results also indicate that the per‐cell EET rate of *S. oneidensis* MR‐1 can be recovered after the oxygen concentration is reduced; however, after about 5 h of operation the per‐cell current production begined to decrease again. Future work is needed to better understand how oxygen impacts EET rates after a well formed biofilm has established on anode electrodes.

The role of riboflavin as electron shuttle varied with DO values. Under the 0.42 mg/L DO conditions, riboflavin showed limited effect on current density and per‐cell EET rate, which might be due to the lower expression of certain *c*‐type cytochrome encoding genes and the subsequent weak interaction of *c*‐type cytochrome proteins with flavins when oxygen is present. Since it is known that oxygen can enhance flavins secretion in *S. oneidensis*, the role of riboflavin may change as a function of oxygen concentration. Further studies are required to better understand the function of flavins under aerobic and anaerobic conditions.

Funding for this research was provided by Synthetic Genomics Inc. (La Jolla, CA, USA) and the Roddenberry Foundation. We also thank Dr. Jeff Gralnick for his useful suggestions.

## Supporting information

Additional supporting information may be found in the online version of this article at the publisher's web‐site.

Supporting Information.
