# Supplementary material for: Effect of oxygen on the per‐cell extracellular electron transfer rate of Shewanella oneidensis MR‐1 explored in bioelectrochemical systems
Source: Biotechnol Bioeng. 2016 Jul 21;114(1):96–105. doi: 10.1002/bit.26046 (PMC5132103; doi:10.1002/bit.26046)
Supplement: Supplementary file 1 — Supporting Information. [file BIT-114-96-s001.docx]

**Supplemental Information:**

Mengqian Lu^1^, Shirley Chan^1^, Sofia Babanova^1,2^, Orianna Bretschger^1,^*

^1^Department of Microbial and Environmental Genomics, J. Craig Venter Institute. 4120 Capricorn Lane, La Jolla, CA, 92037

^2^Chemical and Biological Engineering, University of New Mexico, Albuquerque, NM 87131

*[obretschger@jcvi.org](mailto:obretschger@jcvi.org)

**1. Total Cell Number**

**Figure S1**. Based on the real time LSC microscopy images, the total cell number in (a) the opti-MFC and (b) the opti-BES can be estimated. The total cell numbers remain relatively constant under the anaerobic condition (red triangle) and continuously increase with DO at 0.42 mg/L (purple square). (c) The total cell number when the current densities reached maximum values at certain riboflavin concentration.

**2. Oxygen Promotes Biomass Growth**

**Figure S2**. Laser scanning confocal (LSC) microscopy images of GFP-expressing MR-1 electrodes at 120 hours. There are obviously more biomass development with DO at 1 mg/L than that in the anaerobic condition.

**3. Evaluation of GFP Fluorescence under Anaerobic Condition**

**Figure S3**. (a) LSC microscopy image of *S. oneidensis* MR-1 on electrode under anaerobic condition at 24 hours. (b) After the anaerobic experiment, the cells were stained with Sybr green and ConA and imaged again. No obvious increase of biomass was observed using Sybr green compared to that using GFP.
